# A global dataset for the production and usage of cereal residues in the period 1997–2021

**DOI:** 10.1038/s41597-023-02587-0

**Published:** 2023-10-09

**Authors:** Andrew Smerald, Jaber Rahimi, Clemens Scheer

**Affiliations:** Institute of Meteorology and Climate Research, Atmospheric Environmental Research (IMK-IFU), Karlsruhe Institute of Technology (KIT), Kreuzeckbahnstr. 19, 82467 Garmisch-Partenkirchen, Germany

**Keywords:** Carbon cycle, Element cycles, Agroecology, Agriculture

## Abstract

Crop residue management plays an important role in determining agricultural greenhouse gas emissions and related changes in soil carbon stocks. However, no publicly-available global dataset currently exists for how crop residues are managed. Here we present such a dataset, covering the period 1997–2021, on a 0.5° resolution grid. For each grid cell we estimate the total production of residues from cereal crops, and determine the fraction of residues (i) used for livestock feed/bedding, (ii) burnt on the field, (iii) used for other off-field purposes (e.g. domestic fuel, construction or industry), and (iv) left on the field. This dataset is the first of its kind, and can be used for multiple purposes, such as global crop modelling, including the calculation of greenhouse gas inventories, estimating crop-residue availability for biofuel production or modelling livestock feed availability.

## Background & Summary

Above-ground crop residues, such as the stalks and stover of cereal plants, are an important resource, since they represent more biomass than is harvested as grain and have an embodied energy equal to about 15% of global human primary energy usage^1^ (below-ground residues are not considered here since, for cereals, they are almost never removed from the field). They account for approximately 19% of livestock feed by weight, and are therefore crucial for the production of meat and milk^2,3^. They also play an important role as a domestic fuel, especially in poorer countries. For example, during the mid 1990s in China biomass burning, of which crop residues constituted more than 50%, provided more energy than was derived from oil^4^. Residues left on the field are also an important source of C and N for agricultural soils, helping to maintain or enhance soil organic carbon (SOC) stocks^5–7^, resulting in better retention of nutrients in the soil, reduced sensitivity of plant growth to drought, lower erosion rates and better soil aeration^8^. At the same time, approximately 10% of crop residues are treated as a waste product and burnt on the field^9^. This clears the way for subsequent planting, helps to control pests and diseases and avoids the costs associated with collecting and transporting the residues. However, burning releases particles into the atmosphere that are damaging to human health and contributes to climate change via CH_4_ and N_2_O emissions^10–12^.

The scientific literature contains a variety of suggestions for how a change in crop residue management could address pressing global issues, such as climate change or the loss of naturally vegetated land. These include partial replacement of fossil fuels with bioenergy generated from crop residues^1,13–15^, leaving a higher fraction of crop residues on the field to increase SOC stocks and thus sequester CO_2_ from the atmosphere^16^ and replacing human-edible grains in livestock diets with crop residues, allowing for a reduction in cropland area^17^. Unfortunately, assessing the effectiveness of any of these proposals is hindered by a lack of knowledge about how crop residues are currently being used, leading to an uncertain baseline and making it difficult to determine the trade-offs associated with allocating a higher fraction of crop residues to a specific usage.

**Fig. 1:**
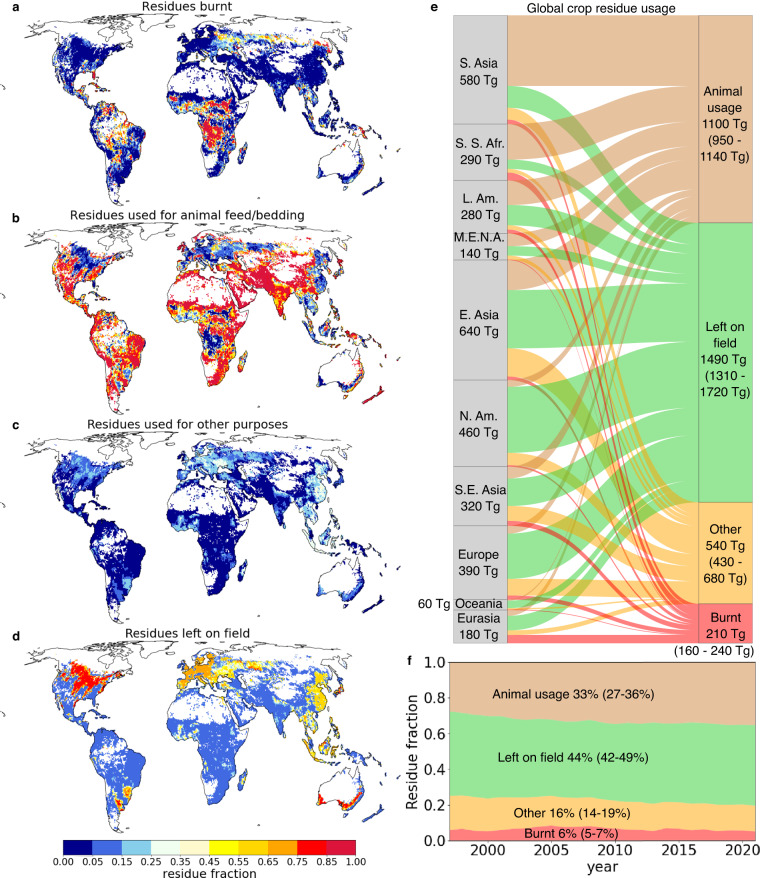
Management of cereal residues in the period 1997–2021. (**a**–**d**) The fraction of residues (**a**) burnt, (**b**) used for livestock, (**c**) used for other off-field purposes, (**d**) and left on the field, averaged over the period and shown on a 0.5° global grid. (**e**) Crop residue production and usage on a regional scale, averaged over the period. Values in brackets show the range of values when taking all 18 calculational schemes into account. (**f**) Timeseries of global crop residue management. The percentages show the average crop residue usage across the period and brackets show the range across the 18 calculational schemes.

Here we address this knowledge gap by constructing a dataset showing crop residue management for cereals on a 0.5° global grid and at an annual timescale for the period 1997–2021. We focus on cereals, since they account for the majority of crop residue production (approximately 73% in 2020^18^), and have a similar energy and protein content, making them relatively uniform in their potential uses^19^. In each grid cell we estimate both crop residue production and the fraction of crop residues that are (1) burnt on the field (2) used for animal feed or bedding (3) used for other off-field purposes such as domestic fuel, industry, mushroom cultivation or construction and (4) left on the field (see Fig. 1). We consider a relatively fine spatial scale, since the high cost of transporting crop residues means that trade-offs between possible management options typically occur at a local level^20^.

We envisage two main uses for the dataset. First, it can be used to improve assessments of the environmental impact of current and possible future agricultural practices, where it has been shown that crop residue management affects greenhouse gas emissions^6,21–26^, emissions of atmospheric pollutants (e.g. NH_3_) and nutrient leaching (e.g. NO_3_^−^)^23,27^. Currently, crop models, which are a common tool for such assessments, typically assume a globally uniform fraction of crop residues left on the field^28–31^, and assess changes in crop residue management relative to this uniform baseline^32–34^ (although some models do have inbuilt ways of estimating crop residue return^35–37^). Incorporating our dataset into regional and global modelling will thus lead to better identification of areas that are hotspots for environmentally harmful losses (e.g. of greenhouse gases) and of areas that would see the greatest benefits from changes in residue management. Second, the dataset can be used to evaluate the trade-offs between different crop residue management options in a spatially explicit way. For example, it can be used to assess the trade-off between soil carbon sequestration, energy generation from biofuel production, and feeding livestock. Currently these trade-offs are either not considered^34,36^ or only considered at country^15^, regional^38^ or global scale^39^.

## Methods

**Fig. 2:**
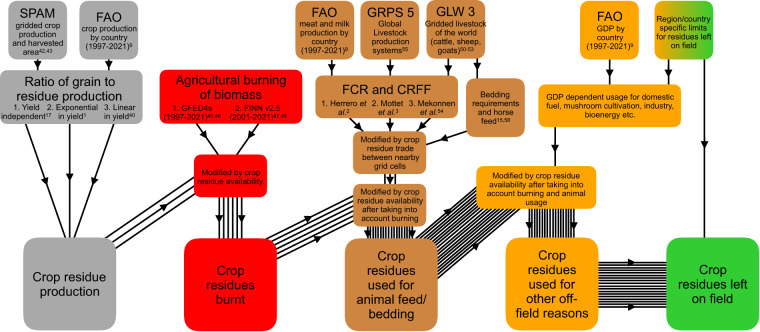
Schematic overview of the calculation of crop residue usage. Output data is shown in the bottom row. Datasets (see Table 1) and data processing steps are coloured according to the output data to which they contribute. Black lines show the number of independent calculations carried through the calculation scheme. Each independent calculation is due to a different combination of input data/methods.

The general strategy employed was to (1) determine crop residue production (2) estimate the quantity of crop residues removed from the field via burning, for animal feed/bedding and other off-field uses and (3) assume the remainder are left on the field after harvest (see Fig. 2). Since multiple methods have been proposed in the literature for determining residue production, biomass burning and livestock feed requirements, we determined a mean value of each of these quantities. This involved averaging three different methods for determining crop residue production, two different datasets for agricultural biomass burning and three different methods for estimating the quantity of crop residues fed to livestock. The same calculational scheme was also used to explore the 18 possible combinations of input data. This resulted in an ensemble of results that allowed us to define a measure of uncertainty (see Fig. 1e,f).

### Cereal residue production

Crop residue production is typically estimated from grain production via harvest indices, which measure the ratio of residue to grain production^1,15,38–41^. Grain production data was taken from the SPAM dataset, which provides gridded, crop-specific production and harvested areas at 5 arcminute resolution^42,43^. This was aggregated to 0.5° resolution and then scaled to match FAO data for yearly, country-specific grain production and harvested areas^9^ (a similar approach was for example used in *Grogan et al*.^44^). The result was a gridded time series for the target period of 1997–2021.

In order to convert grain production to crop residue production, we considered three different methods. (1) *Residue production ratios -* a set of fixed ratios was used to convert grain production to crop residue production. These ratios are crop and region specific, but constant over time^17^. (2) *Exponential yield dependence* - grain yields were converted to crop residue yields using the empirical formula^1^,

$$R=\left\{\begin{array}{cc}aY{e}^{-bY} & for\;Y\le 1/b\\ \frac{a}{b{e}^{1}} & for\;Y > 1/b\end{array}\right.$$

where *R* is the crop residue yield (Mg/ha), *Y* is the grain yield (Mg/ha) and *a* and *b* are crop specific parameters determined by fitting measurement data and shown in Table 2^1,40^. Using a yield-dependent function accounts for variation in the residue production ratio over time, for example due to increasing grain yields. (3) *Linear yield dependence* - grain yields were converted to crop residue yields using the empirical formula,

$$R=Y\left(c-dY\right)$$

where *c* and *d* are crop specific parameters determined by fitting measurement data and shown in Table 2^40^. Finally, crop-specific values for residue production were combined to give total cereal-residue production.

**Table 1:** Global datasets used in the construction of the crop residue usage dataset.

Dataset name	Year(s)	Spatial resolution	Description
Spatial Production Allocation Model (SPAM)^42,43^	2010	5 arcmin global grid	Crop specific production and harvested areas
FAOSTAT^9^	1997–2021	Country	Crop and livestock production, GDP
Global Fire Emissions Database (GFED4s)^45,46^	1997–2021 (monthly)	0.25° global grid	Biomass burning split by vegetation type
The Fire Inventory from NCAR (FINNv2.5)^47,48^	2001–2021	Point based	List of fires, including location, date, biomass burnt and vegetation type
Global Distribution of Ruminant Livestock Production Systems (GRPS 5)^55^	2000s	5 arcmin global grid	Dominant livestock production system in each grid cell.
Gridded Livestock of the World (GLW 3)^50–53,56,57^	2010	5 arcmin global grid	Livestock populations.

**Table 2:** Parameters used to convert grain yields to residue yields.

	a (−)	b (ha/Mg)	c (−)	d (ha/Mg)
Barley	1.822	0.149	2.77	0.27
Maize	2.656	0.103	2.2	0.13
Rice	2.45	0.084	2.56	0.22
Wheat	2.183	0.127	1.96	0.14
Millet	1.9	0.250	4.38	0.95
Sorghum	2.302	0.100	4.55	0.55
Other cereals	1.9	0.250	2.7	0.2

### On-field burning

We considered two data sources for the on-field burning of agricultural biomass: (1) The Global Fire Emissions Database (GFED4s) provides monthly estimates of total agricultural biomass burning at a 0.25° resolution for the period 1997–2021. This is based on a combination of satellite observations and biogeochemical modelling^45,46^; (2) The Fire Inventory from NCAR (FINNv2.5) provides a global list of fires with associated location, date, vegetation-type and total biomass burned for the period 2002–2021^47,48^. In both cases we aggregated the data to determine the total yearly burning of agricultural biomass on a 0.5° global grid. While cereal residues form the dominant type of agricultural residues burnt on a global scale (approximately 96%^9^), other residues can be locally important, especially sugar cane. For example, in Brazil sugar cane accounts for 24% of burnt agricultural biomass^9^. This is partially accounted for by limiting the total biomass burning in each grid cell to be a maximum of 90% of the cereal residue production (see validation section for additional discussion).

### Ruminant feed

Cereal residues are an important feed for ruminants, especially cattle, sheep and goats, but are unpalatable to monogastric animals such as pigs and chickens. As such, there is a clear divide, with most of the residues fed to ruminants derived from cereals, while other important residue classes, such as those from sugarcane and legumes, are predominantly fed to monogastrics^17,49^.

In order to calculate crop residue usage for ruminant feed, country specific data for meat and milk production from cattle, sheep and goats for the period 1997–2021 was obtained from FAOSTAT^9^. The production was then apportioned to grid cells based on the Gridded Livestock of the World (GLW 3) dataset, which gives estimates of animal numbers (including cattle, sheep and goats) for the year 2010^50–53^, resulting in a gridded production time series for 1997–2021. Meat and milk production numbers in each grid cell were transformed into crop residue usage via feed conversion ratios (FCRs) and crop residue feed fractions (CRFFs). FCRs give the quantity of feed (kg-dry-matter) required to produce a given quantity of meat or milk protein at the herd level (i.e. they take into account the need to also feed unproductive animals, such as juveniles). CRFFs give the typical fraction by weight that crop residues make up in an animal’s diet. Regional averages for FCRs and CRFFs have been tabulated in the literature and we use three different sources: (1) *Herrero et al*.^2^ provide FCRs and CRFFs for 10 world regions, and further divide these by animal type (cattle or small ruminant), production type (meat or milk), livestock production system (grazing, mixed, urban or other) and climate (arid, temperate or tropical); (2) *Mekonnen and Hoekstra*^54^ give FCRs for 10 world regions divided by animal type (cattle or small ruminant), production system (grazing, mixed and industrial) and production type (meat or milk, only cattle); (3) *Mottet et al*.^3^ provides FCRs and CRFFs for two world regions (OECD and non-OECD), divided by production system (grazing, mixed or feedlot). Since *Mekonnen and Hoekstra* don’t give CRFFs, we combine their FCRs with the CRFFs of *Herrero et al*. The dominant livestock production system in each 0.5° grid cell is determined from the Global Distribution of Ruminant Livestock Production Systems (GRPS 5) dataset, which documents livestock production systems on a scale of 5 arcminutes, including climate information (arid, temperate or tropical)^55^. Where necessary, the protein content of meat and milk was calculated using 138 g-protein/kg for bovine meat, 137 g-protein/kg for sheep and goat meat and 33 g-protein/kg for milk^2^. The outcome of these calculations was three distinct estimates for crop residue usage by animals. These numbers were further modified by crop residue availability (see below).

### Animal bedding and horse feed

On a global scale, data concerning the use of crop residues for animal bedding is scarce. However, it can form a significant fraction of livestock crop residue usage in richer countries, where residues typically don’t form an important part of ruminant diets^2,3,54^. Here we use estimated bedding requirements for the EU^15^, and apply these on a global scale. Our assumption is that these numbers are reasonable for richer countries, where bedding makes up a higher proportion of livestock residue usage. Taking into account seasonal differences and differences between production systems, we consider a bedding usage of 0.375 kg/day for cattle, 0.1 kg/day for sheep and goats, 1.5 kg/day for horses and 0.0625 kg/day for pigs^15^. These daily requirements are converted to gridded yearly requirements using the GLW 3 dataset for animal populations^50–53,56,57^. For horses we additionally include 420 kg-fresh-matter/year of straw usage for chewing^58^. Crop residue usage for poultry bedding is not considered, since industrial farms typically use other materials, such as sawdust^59^.

### Trading of crop residues

For some grid cells, the estimate of animal residue usage exceeds residue availability. This is unsurprising, since it is known that crop residues are traded for use as animal feed/bedding. For example, in the UK crop residue demand in the livestock-dominated west, is partially met by importing cereal residues from the arable-dominated east^60^. In order to reduce the mismatch between supply and demand, we allow for trade from over- to under-supplied grid cells. We consider a grid cell to be in deficit if the combined on-field burning and animal usage exceed 90% of crop residue production. Deficit grid cells are allowed to import surplus production from nearby grid cells, favouring local supply, but with a maximum distance of 20 grid cells (approximately 1000 km at the equator). The total global deficit before taking trade into account is 270–510 Tg/yr (i.e. the sum of residue deficits across all grid cells in which a deficit exists). After the trade correction this is reduced to 60–100 Tg/yr. Finally, remaining deficits are assumed to arise from a local overestimation of animal usage and are set to zero, meaning that the combination of on-field burning and animal usage never exceeds 90% of production.

### Other off field uses

In poorer countries, a significant fraction of crop residues may be burnt for domestic fuel^4^. The extent of burning largely depends on the availability of other fuels, such as wood, and on competition with the demand for animal feed. In large parts of Africa and South Asia, the high demand for animal feed leaves little available for other off-field uses (see Fig. 1b). However, in China, where livestock production is dominated by monogastrics^9^ and forest cover is low by global standards^9^, crop residues have been historically important as a domestic fuel. In the mid-1990s, when China’s GDP was only marginally above the threshold that the World Bank considers to be low income, *Sinton et al*.^4^ report that approximately 4 EJ of energy was generated by domestic burning of crop residues. Assuming that 18 MJ/kg of energy is released^1^, and that approximately 730 Tg/yr of residues were produced in China at that time, results in 30% of crop residues being used for domestic fuel. We take the figure of 30% as an upper bound for crop residue usage for other off-field purposes in poorer countries (also including construction materials, mushroom cultivation etc.), with the proviso that outside of China a lack of availability means that this boundary is rarely reached (see Fig. 1c and also Supplementary Note 1 for a comparison of the dataset to the results of site-scale farmer surveys in Africa and South Asia). When applying this upper boundary we follow the World Bank in defining poor countries as those with GDP per capita of less than $1046 in 2015 prices.

For rich countries, other off-field uses include usage for industry, construction, the cultivation of mushrooms and strawberries, energy generation via biogas reactors and losses during transport and storage^18^. In Germany it is estimated that these other uses account for approximately 4% of crop residue production (excluding losses)^58^, while in Denmark, which has an exceptionally large biogas generating capacity, they account for 23%^61^. We assume that approximately 10% of crop residues are used for other off field purposes in rich countries, which are defined as those with GDP per capita greater than $12735 at 2015 prices.

For each grid cell, the fraction of other off-field uses is determined by the GDP per capita of the corresponding country, along with crop residue availability. For middle income countries the maximum usage is determined by linearly interpolating between the poor country (30%) and rich country (10%) values according to GDP per capita. Changes in GDP per capita over time thus result in a changing upper bound for other off-field crop residue usage. Residue removal via a combination of on-field burning, animal usage and other usage is capped at 90% of production.

### Crop residues left on field

Crop residues not assigned another usage are assumed to be left on the field. Due to the constraints above, at least 10% of residues are left on the field, and this is imposed due to the difficulty of fully removing residues. In addition, we impose a maximum fraction of crop residues left on the field in a limited number of countries and regions, assigning any excess to other off-field uses. The aim is to further improve the balance between on-field and off-field uses of crop residues, compensating for the lack of global data about crop residue use for domestic fuel, industry, construction, biofuels etc. In China we impose a limit of 60% of residues returned to the field in every grid cell. This results in a good match to literature values, where it was reported that 46% (2009)^62^ and 52% (2019)^63^ of residues were returned to the field. Using the 60% limit our dataset estimates 47% (2009) and 51% (2019), while in the absence of such a limit the figures are 51% (2009) and 60% (2019) (see also Supplementary Note 2). In Europe, it has been estimated that 40–70% of crop residues need to be returned to the field to maintain soil organic carbon and avoid erosion^15^. We thus assume that it is rare for more than 70% of residues to be returned to the field, since this is not necessary for agricultural reasons, and selling excess straw is a way to supplement farm income. In North America and Oceania we impose a higher limit of 80%, since the often semi-arid conditions require a higher percentage of residues to be returned to the field to guard against erosion^64^. For Australia we find good agreement between the estimate of our dataset (74% returned to the field in 2012) and the Australian Bureau of Statistics (76% in 2012)^65^. On a global scale, the imposition of maximum return rates results in 3% of production being shifted from on-field to off-field usage.

## Data Records

The datasets are stored as netCDF files in the RADAR4KIT repository and can be accessed at 10.35097/989^66^. The spatial resolution is a 0.5° global grid and the temporal resolution is annual for the years 1997–2021.

### Main dataset

Values obtained with mean input values are intended as the default dataset. The filename is:

crop_residue_usage_mean.nc

### Additional datasets

There are 18 additional datasets, corresponding to all possible method combinations (see Methods section above)^66^. The naming convention is:

crop_residue_usage_{*residue_production_ratio*}_{*livestock_source*}_{*fire_dataset*}.nc

where:

{*residue_production_ratio*} = constant, exponential, linear

{*livestock_source*} = herrero, mottet, mekonnen

{*fire_dataset*} = GFED4s, FINN

Each dataset has 5 layers and all units are Mg/year:

1. *residue_production*:- crop residue production from cereals
2. *burnt_residues*:- residues burnt on the field
3. *animal_usage*:- residues used for livestock feed and bedding
4. *other_usage*:- residues used for other off-field purposes (domestic fuel, construction…)
5. *left_on_field*:- residues left on the field

For each grid cell and year the residue production (1) is equal to the sum of (2), (3), (4) and (5), meaning that all residues are assigned a use. Fractional residue usage is simply calculated by dividing the specific residue usage by the residue production.

## Technical Validation

In order to validate our dataset, we have collected literature values for cereal residue production and fractional residue usage. We only considered studies that (1) fall within the period 1997–2021, (2) report at a country level or higher and (3) include all major cereals grown within the country/region.

**Fig. 3:**
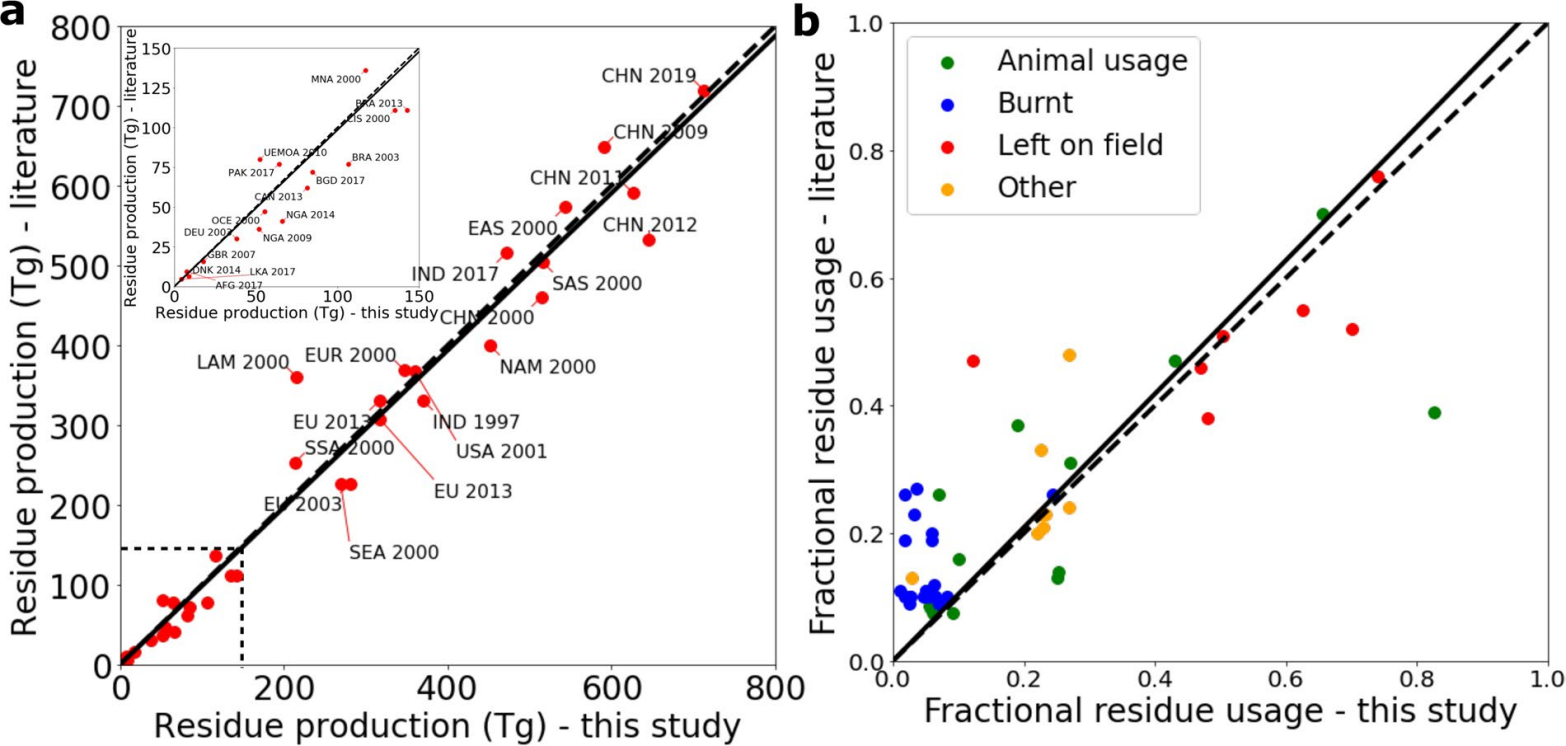
Comparison to literature values for residue production and fractional residue usage. (**a**) Cereal residue production at country and regional level (see Supplementary Table 1). The solid line is a linear regression fit (r^2^ = 0.96) and the dashed line a 1:1 line. (**b**) Fractional usage of cereal residues at country and regional level (see Supplementary Table 2). The point colour shows the usage type, the solid line is a linear regression fit (r^2^ = 0.70) and the dashed line a 1:1 line.

For crop residue production there is close agreement between our estimate and literature values. On a global scale *Smil et al*.^67^ estimated 2500 Tg of cereal residue production in 1997, *Lal et al*.^13^ 2800 Tg in 2001 and *Shinde et al*.^18^ 3860 Tg in 2020. These values can be compared with our estimates of 2930, 2930 and 3900 Tg in the respective years. At the country and regional scale, Fig. 3a shows a comparison between this study and literature^11,13,15,38,40,41,60–63,68–75^. Linear regression analysis gives a coefficient of best fit of 0.98 with r^2^ = 0.96.

Data concerning fractional crop-residue usage is even sparser than that for crop-residue production. A linear regression analysis comparing our study with literature values^9,11,40,60–63,65,68–71,73,75–80^ results in a coefficient of best fit of 1.05 and r^2^ = 0.70 (see Fig. 3b), suggesting good agreement.

One point that stands out in Fig. 3b is that burnt fractions are typically higher in the literature than in our study. For example, on a global scale the FAO estimates that 10–11% of rice, wheat and maize residues are burnt^9^, while we find 5–8%. Literature estimates for China range from 9%^9^ to 27%^68^ and for India from 9%^9^ to 20%^79^. This can be compared to our estimates, derived from satellite data, of 2–5% in China and 5–7% in India. It is not clear whether this discrepancy results from satellite data not capturing all fires or from methodological problems with other approaches, such as farmer surveys^78^.

### Data gaps

There is significant scope to further improve our estimates of crop residue usage via improvements in the input data. Here we discuss which improvements would have the highest priority.

#### Crop residue production

Estimates of crop residue production fit well with the literature (see Fig. 3), but could be further improved by better spatial and temporal resolution (harvest indices) or fitting empirical functions to larger measurement datasets (exponential and linear approaches). However, this is not a high priority for improving our crop residue dataset.

#### Animal usage

Improving the spatial and temporal resolution of residue feed fractions and feed conversion ratios is the most obvious way to improve estimates of crop residue use for livestock feed. Since this is a significant use of crop residues, especially in poorer countries, this would better constrain what fraction of crop residues are left for all other uses.

#### Burning

As discussed above, literature values for agricultural biomass burning vary significantly between those derived from satellite data and those from other sources (which themselves have significant variation). Finding a way to improve the consistency of these estimates would be a priority.

#### Other off field uses

There is a lack of global data for crop residue usage for domestic fuel, construction, biofuels etc. The existence of such a dataset would significantly improve our estimates of crop residue management.

#### Left on field

The fraction of residues left on the field also lacks global data. However, even in the absence of such a dataset, improvements in data for off-field usage would allow on-field usage to be better constrained, and would remove the need for imposing minimum and maximum limits.

### Supplementary information

Supplementary Information

## Data Availability

The codes used to generate the dataset are available at https://zenodo.org/record/7843730#.ZEIxAC0Rq3I^81^.
