# Supplementary material for: A global dataset for the production and usage of cereal residues in the period 1997–2021
Source: Sci Data. 2023 Oct 9;10:685. doi: 10.1038/s41597-023-02587-0 (PMC10562449; doi:10.1038/s41597-023-02587-0)
Supplement: Supplementary file 1 — Supplementary Information [file 41597_2023_2587_MOESM1_ESM.pdf]

Supplementary Information

Table of Contents

p2 - Supplementary Note 1: Crop residue usage in Sub-Saharan Africa and South Asia

p3 - Supplementary note 2: Setting an upper bound for crop residue return in China

p4 - Supplementary Table 1: Validation data for crop residue production

p5 - Supplementary Table 2: Validation data for fractional crop residue use

Supplementary Note 1: Crop residue usage in Sub-Saharan Africa and South Asia

One of the few papers to give a complete breakdown of crop residue usage across multiple sites is Valbuena *et al.*,[1], who performed farmer surveys in 2010 for specific regions of Sub-Saharan Africa and South Asia. Here we compare our dataset to their findings. Since Valbuena *et al.* considered clusters of villages surrounding a local market at distances of up to 50km, we compared their results to a small cluster of grid cells (specifically a square of 9 grid cells surrounding the location of the market). As can be seen in Figure 1, there is reasonable agreement between our dataset and the findings of Valbuena *et al.*

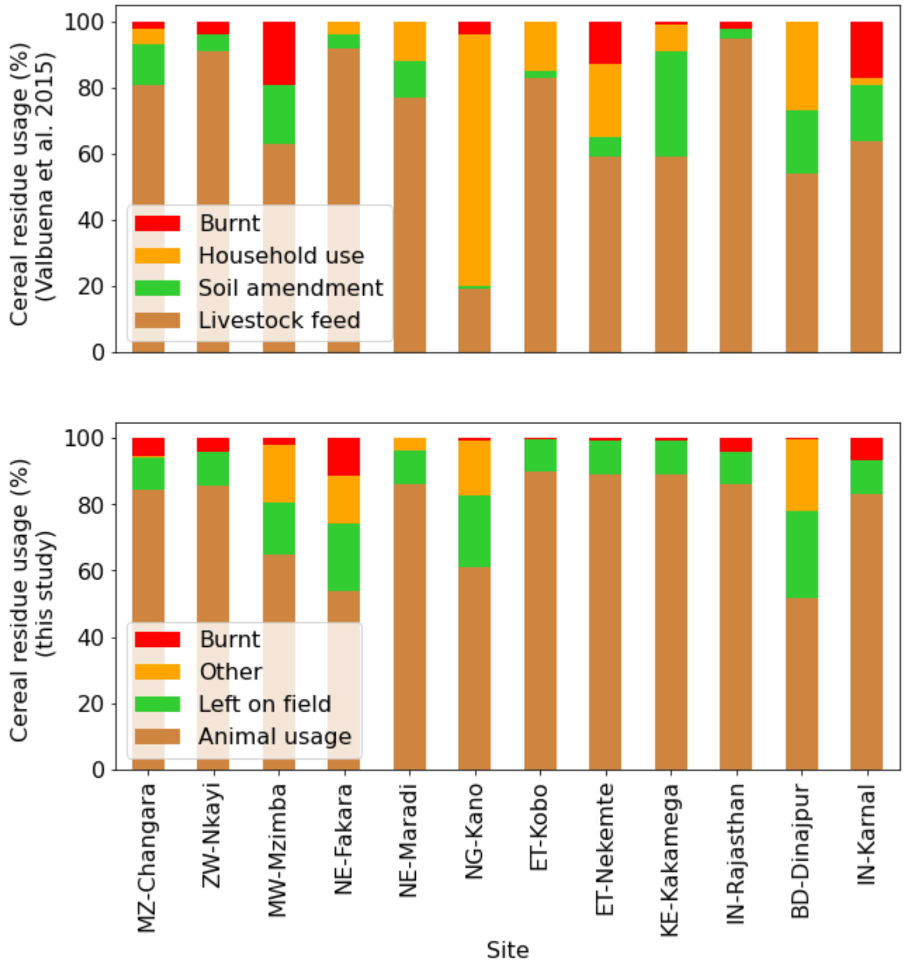

Supplementary Figure 1: Comparison of crop residue usage between our dataset and the survey results of Valbuena *et al.* Household survey results for crop residue usage in the year 2010 were reported by Valbuena et al. for 12 sites across 9 countries in Sub-Saharan Africa (MZ=Mozambique, ZW=Zimbabwe, MW=Malawi, NE=Niger, NG=Nigeria, ET=Ethiopia and KE=Kenya) and South Asia (IN=India and BD=Bangladesh)[1]. These are compared to the corresponding grid cells in our dataset.

Supplementary Note 2: Setting an upper bound for crop residue return in China

Here we consider two reports of crop residue management in China that give a full breakdown of the different usages. These include Jiang *et al.*[2], which reports data from the Chinese Ministry of Agriculture for 2009, and Li *et al.*[3], which reports data for 2019. We compare these results to our dataset, and use them to justify the choice of an upper boundary of residues left on the field of 60% on a grid cell basis. The imposition of an upper boundary redistributes crop residue usage from being left on the field to other off-field uses, while leaving livestock and burning unaffected.

Jiang *et al*. reports that 46% of residues were used as a soil amendment in 2009 and Li *et al*. report 52% in 2019. Using an upper boundary of 60% of residues left on the field for grid cells in China results in the corresponding values being 47% and 51% (see Figure 2). In the absence of a boundary the values would be 51% and 60%.

It should be noted that there are some question marks concerning the allocation of crop residues reported in the two papers. Jiang *et al*. do not report any burning of crop residues, which contradicts satellite data. Li *et al*. report that 16% of crop residues are not used productively, but do not state what happens to these residues. In Figure 2 we have assigned these to burning, but it could be that some of them are lost in harvesting, transport or storage, which would instead fit into our other off-field use category. As such, the relatively poor fit between the burning fraction shown for 2019 in Figure 2 may be due to a misrepresentation of Li *et al*.'s data. It is also worth noting that both papers show data for all crop residues, including non-cereal crops. According to Li *et al*. cereal crops account for more than 83% of total residue production, suggesting that the comparison is valid, but differential usage between cereal and non-cereal crops may still have a small effect on the comparison to our dataset.

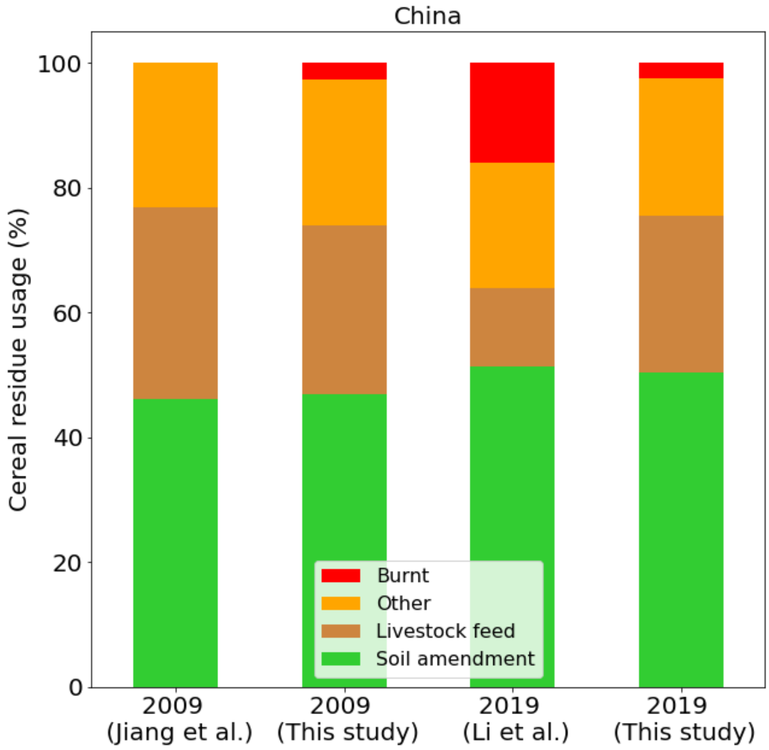

Supplementary Figure 2: Comparison of crop residue usage in China in 2009 and 2019 between our dataset and the papers of Jiang *et al.*[2] and Li *et al.*[3]

Supplementary tables

Reference	Country/region	Year	Ref. Value (Tg)	Dataset value (Tg)
Lal[4]	USA	2001	367	360
Li *et al*.[5]	Canada	2013	62	65
Krausmann *et al*.[6]	N. America	2000	400	452
Li *et al*.[3]	China	2019	720	712
Jiang *et al*.[2]	China	2009	649	590
Zhang *et al*.[7]	China	2011	591	626
Krausmann *et al*.[6]	E. Asia	2000	574	544
Li *et al*.[8]	China	2012	532	645
Cao *et al*.[9]	China	2000	461	515
SAARC[10]	India	2017	516	472
SAARC[10]	Pakistan	2017	77	64
SAARC[10]	Bangladesh	2017	72	85
SAARC[10]	Afghanistan	2017	9.4	8.7
SAARC[10]	Sri Lanka	2017	4.5	4.3
Krausmann *et al*.[6]	S. Asia	2000	504	516
Ravindranath *et al*.[11]	India	1997	331	370
Krausmann *et al*.[6]	Europe	2000	369	347
Garcia *et al*.[12]	EU	2013	331	317
Ronzon *et al*.[13]	EU	2013	308	317
Scarlat *et al*.[14]	EU	2003	227	281
Weiser *et al*.[15]	Germany	2003	30	38
Copeland *et al*.[16]	UK	2007	15.7	17.9
IEA bioenergy[17]	Denmark	2014	6.2	8.8
Krausmann *et al*.[6]	Latin America	2000	360	216
Cherubin *et al*.[18]	Brazil	2003	77	107
Cherubin *et al*.[18]	Brazil	2013	111	143
Krausmann *et al*.[6]	Sub-Saharan Africa	2000	253	204
FAO W. Afr.[19]	UEMOA (W. Africa)	2010	80	53
Ayodele *et al*.[20]	Nigeria	2009	36	52
Ayodele *et al*.[20]	Nigeria	2014	41	66
Krausmann *et al*.[6]	S.E. Asia	2000	226	270
Krausmann *et al*.[6]	M.E.N.A.	2000	136	117
Krausmann *et al*.[6]	Eurasia	2000	111	135
Krausmann *et al*.[6]	Oceania	2000	47	55

Supplementary Table 1: Validation data for crop residue production.

Reference	Usage	Country/region	Year	Ref. fraction	Dataset fraction
Ravindranath *et al.*[11]	Animal	India	1997	0.47	0.43
Weiser *et al.*[15]	Animal	Germany	2003	0.16	0.10
IEA bioenergy[17]	Animal	Denmark	2014	0.26	0.07
Zhang *et al.*[7]	Animal	China	2011	0.14	0.25
Li *et al.*[5]	Animal	Canada	2001	0.08	0.06
Li *et al.*[5]	Animal	Canada	2006	0.09	0.06
Li *et al.*[5]	Animal	Canada	2010	0.08	0.06
Copeland *et al.*[16]	Animal	UK	2007	0.37	0.19
Li *et al.*[3]	Animal	China	2019	0.13	0.25
FAO W. Afr.[19]	Animal	Mali	1997	0.70	0.66
FAO W. Afr.[19]	Animal	Niger	1998	0.39	0.83
Ronzon *et al.*[13]	Animal	EU	2013	0.08	0.09
Jiang *et al.*[2]	Animal	China	2009	0.31	0.27
Cao *et al.*[9]	Burnt	China	2000	0.26	0.02
Li *et al.*[8]	Burnt	China	2012	0.23	0.03
FAOSTAT[21]	Burnt	China	2000	0.10	0.02
FAOSTAT[21]	Burnt	China	2005	0.10	0.03
FAOSTAT[21]	Burnt	China	2010	0.10	0.03
FAOSTAT[21]	Burnt	China	2015	0.10	0.05
FAOSTAT[21]	Burnt	China	2020	0.09	0.02
FAOSTAT[21]	Burnt	World	2000	0.11	0.05
FAOSTAT[21]	Burnt	World	2005	0.10	0.08
FAOSTAT[21]	Burnt	World	2010	0.10	0.07
FAOSTAT[21]	Burnt	World	2015	0.10	0.06
FAOSTAT[21]	Burnt	World	2020	0.10	0.06
FAOSTAT[21]	Burnt	India	2000	0.11	0.01
FAOSTAT[21]	Burnt	India	2005	0.11	0.05
FAOSTAT[21]	Burnt	India	2010	0.10	0.05
FAOSTAT[21]	Burnt	India	2015	0.10	0.06
FAOSTAT[21]	Burnt	India	2020	0.09	0.07
Sharma *et al.*[22]	Burnt	India	2019	0.12	0.06
Zhang *et al.*[7]	Burnt	China	2011	0.27	0.04
Huang *et al.*[23]	Burnt	India	2014	0.10	0.06
Yan *et al.*[24]	Burnt	China	2000	0.19	0.02
Devi *et al.*[25]	Burnt	India	2014	0.19	0.06
FAO W. Afr[19]	Burnt	Mali	1997	0.26	0.24
FAO W. Afr[19]	Left on field	Niger	1998	0.47	0.12
IEA bioenergy[17]	Left on field	Denmark	2014	0.52	0.70
Zhang *et al.*[7]	Left on field	China	2011	0.38	0.48
ABS[26]	Left on field	Australia	2012	0.76	0.74
Li *et al.*[3]	Left on field	China	2019	0.51	0.50
Jiang *et al.*[2]	Left on field	China	2009	0.46	0.47
Townsend *et al.*[27]	Left on field	UK	2012	0.55	0.63
Ravindranath *et al.*[11]	Other off-field	India	1997	0.33	0.23
IEA bioenergy[17]	Other off-field	Denmark	2014	0.23	0.23
Cao *et al.*[9]	Other off-field	China	2000	0.48	0.27
Zhang *et al.*[7]	Other off-field	China	2011	0.21	0.23

Li *et al.*[3]	Other off-field	China	2019	0.20	0.22
Jiang *et al.*[2]	Other off-field	China	2009	0.23	0.23
Yan *et al.*[24]	Other off-field	China	2000	0.24	0.27
FAO W. Afr.[19]	Other off-field	Niger	1998	0.13	0.03

Supplementary Table 2: Validation data for fractional crop residue use.

1. Valbuena, D. *et al.* Identifying determinants, pressures and trade-offs of crop residue use in mixed smallholder farms in Sub-Saharan Africa and South Asia. *Agric. Syst.* **134**, 107–118 (2015).
2. Jiang, D., Zhuang, D., Fu, J., Huang, Y. & Wen, K. Bioenergy potential from crop residues in China: Availability and distribution. *Renew. Sustain. Energy Rev.* **16**, 1377–1382 (2012).
3. Xinxin, L., Zuliang, S., Jiuchen, W. & Rongfeng, J. Review on the Crop Straw Utilization Technology of China. *Am. J. Environ. Sci. Eng.* **4**, 61–64 (2020).
4. Lal, R. World crop residues production and implications of its use as a biofuel. *Environ. Int.* **31**, 575–584 (2005).
5. Li, X. *et al.* A review of agricultural crop residue supply in Canada for cellulosic ethanol production. *Renew. Sustain. Energy Rev.* **16**, 2954–2965 (2012).
6. Krausmann, F., Erb, K.-H., Gingrich, S., Lauk, C. & Haberl, H. Global patterns of socioeconomic biomass flows in the year 2000: A comprehensive assessment of supply, consumption and constraints. *Ecol. Econ.* **65**, 471–487 (2008).
7. Zhang, G. *et al.* Residue usage and farmers' recognition and attitude toward residue retention in China's croplands. *J. Agro-Environ. Sci.* **36**, 981–988 (2017).
8. Li, J., Bo, Y. & Xie, S. Estimating emissions from crop residue open burning in China based on statistics and MODIS fire products. *J. Environ. Sci.* **44**, 158–170 (2016).
9. Cao, G., Zhang, X. & Zheng, F. Inventory of black carbon and organic carbon emissions from China. *Atmos. Environ.* **40**, 6516–6527 (2006).

10. *Possible uses of crop residue for energy generation instead of open burning*. (2021).

11. Ravindranath, N. H. *et al.* Assessment of sustainable non-plantation biomass resources potential for energy in India. *Biomass Bioenergy* **29**, 178–190 (2005).

12. García-Condado, S. *et al.* Assessing lignocellulosic biomass production from crop residues in the European Union: Modelling, analysis of the current scenario and drivers of interannual variability. *GCB Bioenergy* **11**, 809–831 (2019).

13. Ronzon, T. & Piotrowski, S. Are primary agricultural residues promising feedstock for the European bioeconomy? *Industrial Biotechnology* vol. 13 113–127 (2017).

14. Scarlat, N., Martinov, M. & Dallemand, J.-F. Assessment of the availability of agricultural crop residues in the European Union: Potential and limitations for bioenergy use. *Waste Manag.* **30**, 1889–1897 (2010).

15. Weiser, C. *et al.* Integrated assessment of sustainable cereal straw potential and different straw-based energy applications in Germany. *Appl. Energy* **114**, 749–762 (2014).

16. Copeland, J. & Turley, D. *National and regional supply/demand balance for agricultural straw in Great Britain*. (2008).

17. IEAbioenergy. *Mobilisation of agricultural residues for bioenergy and higher value bio-products: Resources, barriers and sustainability*. https://www.ieabioenergy.com/wp-content/uploads/2018/01/TR2017-01-F.pdf (2017).

18. Cherubin, M. R. *et al.* Crop residue harvest for bioenergy production and its implications on soil functioning and plant growth: A review. *Sci. Agric.* **75**, (2018).

19. Gouro, A. & Ly, C. *Crop residues and agro-industrial by-products in West Africa*. (FAO Regional Office for Africa, 2014).

20. Ayodele, O. P. & Aluko, O. A. Weed Management Strategies for Conservation Agriculture and Environmental Sustainability in Nigeria. *IOSR J. Agric. Vet. Sci. IOSR-JAVS* **10**, 1 (2017).
21. FAO. FAOSTAT. Rome: Food and Agriculture Organization of the United Nations. (2023).
22. Sharma, D. Crop Residue Management- Challenges and Solutions: A New Paradigm in Agriculture. *Agric. Environ. E Newsl.* **1**, 103 (2020).
23. Huang, T. *et al.* Health and environmental consequences of crop residue burning correlated with increasing crop yields midst India's Green Revolution. *Npj Clim. Atmospheric Sci.* **5**, 81 (2022).
24. Yan, X., Ohara, T. & Akimoto, H. Bottom-up estimate of biomass burning in mainland China. *Atmos. Environ.* **40**, 5262–5273 (2006).
25. Devi, S., Gupta, C., Jat, S. L. & Parmar, M. S. Crop residue recycling for economic and environmental sustainability: The case of India. *Open Agric.* **2**, 486–494 (2017).
26. *Land management and farming in australia, 2011-12*. https://www.abs.gov.au/ausstats/abs@.nsf/Lookup/4627.0main+features62011-12.
27. Townsend, T. J., Sparkes, D. L., Ramsden, S. J., Glithero, N. J. & Wilson, P. Wheat straw availability for bioenergy in England. *Energy Policy* **122**, 349–357 (2018).
